# Performance of fully automated deep-learning-based coronary artery calcium scoring in ECG-gated calcium CT and non-gated low-dose chest CT

**DOI:** 10.1007/s00330-025-11559-4

**Published:** 2025-05-10

**Authors:** Sihwan Kim, Eun-Ah Park, Chulkyun Ahn, Baren Jeong, Yoon Seong Lee, Whal Lee, Jong Hyo Kim

**Affiliations:** 1 Department of Applied Bioengineering, Graduate School of Convergence Science and Technology, Seoul National University, Seoul, Republic of Korea; 2 ClariPi Research, Seoul, Republic of Korea; 3 Department of Radiology, Seoul National University Hospital, Seoul, Republic of Korea; 4 Department of Radiology, Seoul National University College of Medicine, Seoul, Republic of Korea; 5 Institute of Radiation Medicine, Seoul National University Medical Research Center, Seoul, Republic of Korea

**Keywords:** Coronary artery calcification, Calcium scoring, Calcium CT, Low-dose chest CT, Deep learning

## Abstract

**Objectives:**

This study aimed to validate the agreement and diagnostic performance of a deep-learning-based coronary artery calcium scoring (DL-CACS) system for ECG-gated and non-gated low-dose chest CT (LDCT) across multivendor datasets.

**Materials and methods:**

In this retrospective study, datasets from Seoul National University Hospital (SNUH, 652 paired ECG-gated and non-gated CT scans) and the Stanford public dataset (425 ECG-gated and 199 non-gated CT scans) were analyzed. Agreement metrics included intraclass correlation coefficient (ICC), coefficient of determination (*R*²), and categorical agreement (κ). Diagnostic performance was assessed using categorical accuracy and the area under the receiver operating characteristic curve (AUROC).

**Results:**

DL-CACS demonstrated excellent performance for ECG-gated CT in both datasets (SNUH: *R*² = 0.995, ICC = 0.997, κ = 0.97, AUROC = 0.99; Stanford: *R*² = 0.989, ICC = 0.990, κ = 0.97, AUROC = 0.99). For non-gated CT using manual LDCT CAC scores as a reference, performance was similarly high (*R*² = 0.988, ICC = 0.994, κ = 0.96, AUROC = 0.98–0.99). When using ECG-gated CT scores as the reference, performance for non-gated CT was slightly lower but remained robust (SNUH: *R*² = 0.948, ICC = 0.968, κ = 0.88, AUROC = 0.98–0.99; Stanford: *R*² = 0.949, ICC = 0.948, κ = 0.71, AUROC = 0.89–0.98).

**Conclusion:**

DL-CACS provides a reliable and automated solution for CACS, potentially reducing workload while maintaining robust performance in both ECG-gated and non-gated CT settings.

**Key Points:**

***Question*** *How accurate and reliable is deep-learning-based coronary artery calcium scoring (DL-CACS) in ECG-gated CT and non-gated low-dose chest CT (LDCT) across multivendor datasets?*

***Findings*** *DL-CACS showed near-perfect performance for ECG-gated CT. For non-gated LDCT, performance was excellent using manual scores as the reference and lower but reliable when using ECG-gated CT scores.*

***Clinical relevance*** *DL-CACS provides a reliable and automated solution for CACS, potentially reducing workload and improving diagnostic workflow. It supports cardiovascular risk stratification and broader clinical adoption, especially in settings where ECG-gated CT is unavailable.*

**Graphical Abstract:**

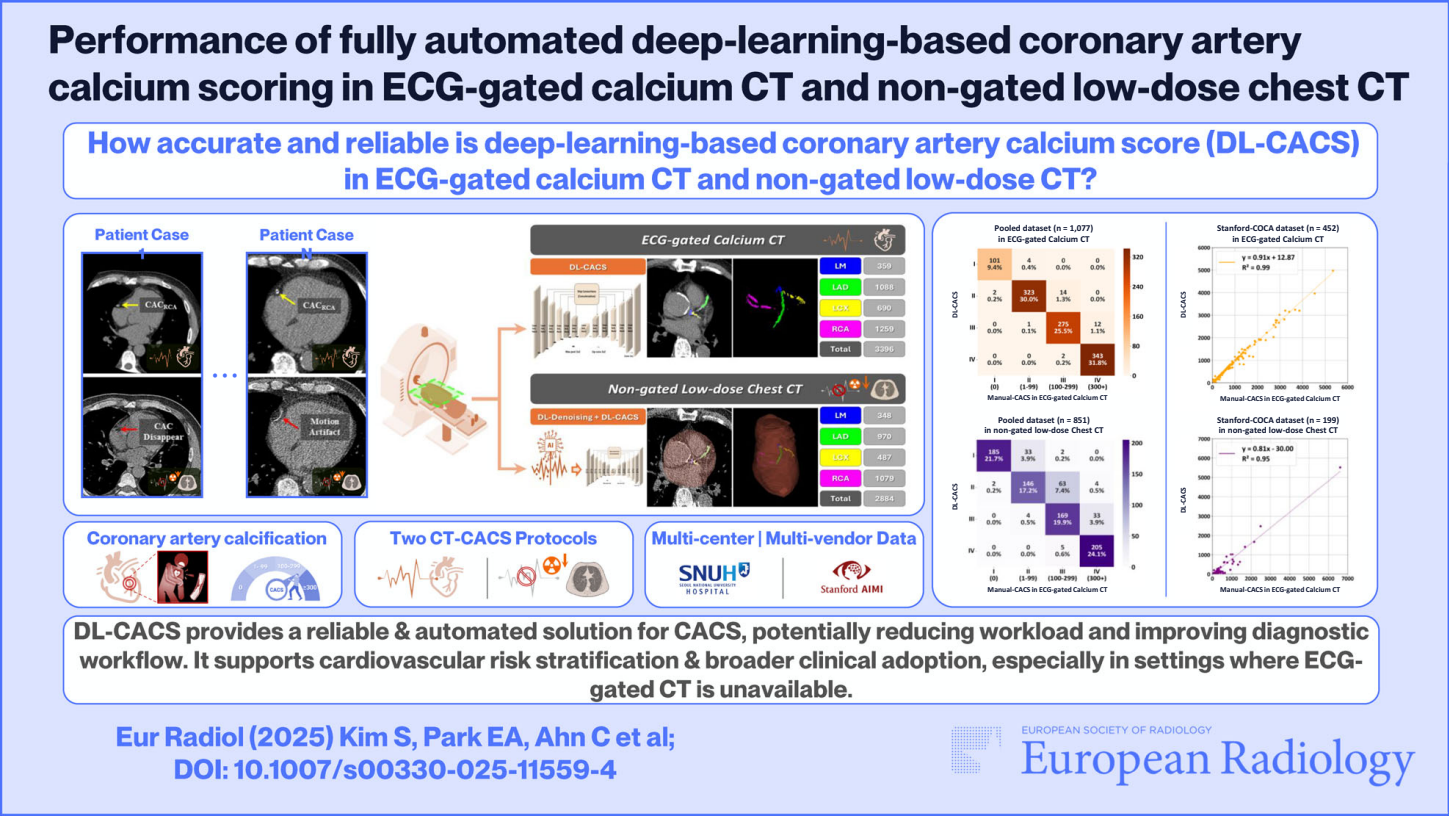

## Introduction

Coronary artery calcium (CAC), a well-established imaging biomarker for coronary atherosclerosis, provides insight into the presence and extent of calcified plaques within the coronary arteries, further enhancing the prediction of future cardiovascular events and improving risk stratification in adjunction with traditional risk factors [1–3]. The 2018 American College of Cardiology/American Heart Association guidelines provide class IIa recommendations for using CACS to manage statin therapy in asymptomatic individuals with borderline or intermediate 10-year atherosclerotic cardiovascular disease (ASCVD) risk [4]. This approach prevents unnecessary treatment and ensures that high-risk individuals receive aggressive interventions, thereby reducing costs and maximizing therapeutic benefits.

The significance of CAC as a risk modifier has underscored the potential of low-dose chest CT (LDCT) scans to detect calcium presence. According to guidelines from the Society of Cardiovascular Computed Tomography and the Society of Thoracic Radiology, visual grading of CAC from LDCT scans should be documented as a class I recommendation [5]. Although the LDCT visual scoring method has demonstrated good concordance with the Agatston score, this concordance is moderate, with kappa values of 0.66–0.75 and accuracies of 56–73% in risk classification, and remains subject to inter-reader variability [6, 7]. Another alternative, manual quantification, may provide more precise measurements but is time-intensive and further complicated by quantum noise and motion artifacts [8, 9]. Advanced artificial intelligence algorithms now enable more precise and efficient quantification, offering clinicians improved diagnostic accuracy, streamlined workflow, and an elevated standard of patient care [10, 11]. Therefore, this study aimed to validate and evaluate the performance of a fully automated, DL-based CACS system on both gated calcium CT and non-gated LDCT across multivendor datasets.

## Materials and methods

### Dataset

**Fig. 1:**
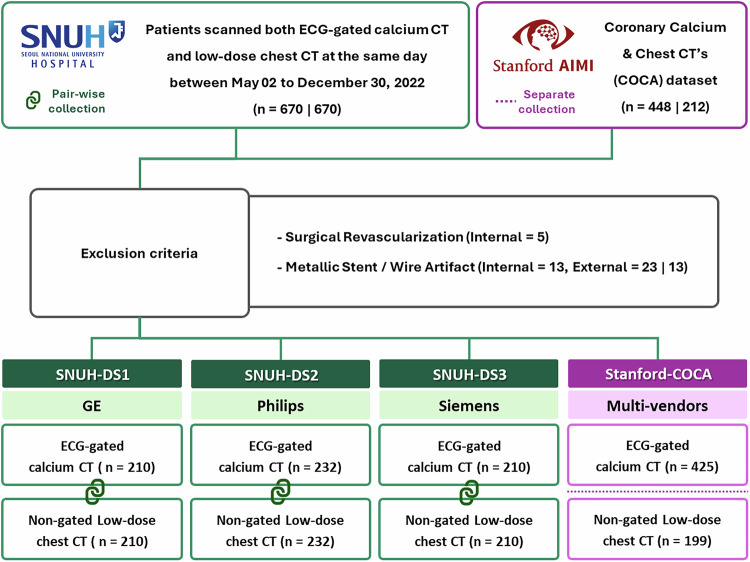
Study flowchart. The flow diagram shows the number of patients undergoing electrocardiogram (ECG)-gated calcium CT and non-gated low-dose chest CT in the Seoul National University Hospital dataset 1–3 (SNUH-DS1–3) and the Stanford-coronary calcium analysis (Stanford-COCA) dataset

The institutional review board approved the study, and the requirement for informed consent was waived owing to the study’s retrospective nature (IRB No. 2305-084-1431). For the clinical performance validation of the DL-CACS model, CAC scanning datasets from two institutions were used (Fig. 1): 652 cases from Seoul National University Hospital (SNUH) and the Stanford coronary calcium and chest CT (Stanford-COCA) dataset, which included 425 ECG-gated calcium CT and 199 non-gated LDCT cases. In the SNUH dataset, cases with surgical revascularization (*n* = 5) and metallic stent or wire artifacts (*n* = 49) were excluded based on visual assessment during manual CAC contouring. The final dataset included 652 subjects (518 males, 134 females; mean age, 60.8 years) who underwent paired ECG-gated calcium CT and non-gated LDCT scans on the same day, using GE (32%), Philips (36%), or Siemens (32%) machines between May and December 2022. In the Stanford-COCA dataset, most LDCT scans were acquired on GE (40%) and Siemens (57%) machines. For ECG-gated coronary calcium CT scans, Siemens machines were predominantly used (99%), with GE machines representing only 1%. The ECG-gated calcium CT scans were specifically acquired for CAC evaluation, while the non-gated LDCT scans were primarily intended for lung cancer screening.

### CT imaging protocols

Detailed CT scan parameters are listed in Table 1. In the SNUH dataset, all ECG-gated calcium CT scans, except those in SNUH-DS3, used 120 kV, 2.5 or 3 mm slice thickness, and standard reconstruction kernels. For SNUH-DS3, Siemens machines utilized 100 kV with a tin filter to reduce radiation and the Sa36f kernel to mitigate kV level effects, though this kernel was not applied to non-gated LDCT scans [12]. Slice thickness for non-gated LDCT reconstruction ranged from 1.0 to 1.25 mm, and iterative reconstruction was used in all SNUH datasets.

**Table 1:** CT acquisition protocol and image reconstruction settings

Acquisition protocol	Dataset	No. of patients	Scanner model	kV	mAs	Exposure time (ms)	Slice thickness (mm)	Reconstruction kernel setting
SNUH datasets: pairwise data (*n* = 652)								
ECG-gated calcium CT	SNUH-DS1	210	GE Revolution	120	75 ± 11	280	2.5	Standard
	SNUH-DS2	232	Philips iCT 256	120	70 ± 75	219	2.5	CB
	SNUH-DS3	210	Siemens Force	Sn100^a^	243 ± 60	148	3.0	Sa36f
Non-gated low-dose chest CT	SNUH-DS1	210	GE Revolution	120	25 ± 4	350	1.25	Standard
	SNUH-DS2	232	Philips iCT 256	120	20 ± 2	666	1.0	YA, C
	SNUH-DS3	210	Siemens Force	Sn100^a^	138 ± 41	500	1.0	Br59(3)
Stanford datasets: independent data (*n* = 624)								
ECG-gated calcium CT	Stanford-COCA^b^	425	GE LightSpeed, GE Discovery, Siemens Force, Siemens Definition	120^c^	109 ± 49	181 ± 58	3.0 ± 0.1	I30f(3), Qr36d(2), Standard
Non-gated low-dose chest CT	Stanford-COCA^b^	199	GE LightSpeed, GE Discovery, GE Revolution, Siemens Definition, Siemens Force, Siemens Sensation, Philips Brilliance, Toshiba Aquilion, etc.	118 ± 8	135 ± 134	437 ± 215	4.9 ± 0.5	I26f(2), I30f(2), I31f(2), I31f(3) B30f(2), B31f(2), Br40s(2), Br40d(2), Standard, FC01, FC12

^a^ Sn100, 100 kV with tin filter attached

^b^ COCA, coronary calcium and chest CT dataset

^c^ 120 kV was used in all cases except one, which used 100 kV

In independent validation using Stanford-COCA dataset, 120 kV was used for all ECG-gated CT cases except for one (100 kV). For non-gated LDCT, 120, 100, and 140 kV were used in 166, 28, and 5 cases, respectively. All ECG-gated CT images had a 3 mm slice thickness, whereas non-gated LDCT reconstruction varied from 0.7 to 5.0 mm. Non-gated LDCT scans utilized both standard and sharp kernels, with iterative reconstruction applied in most scans.

### CACS quantification as the reference

In the SNUH dataset, an experienced technologist, under the supervision of a radiologist with over 20 years of experience, semi-manually calculated the CACS in ECG-gated calcium CT using semi-automated software (Syngovia, Siemens Healthineers). Calcified regions were identified as contiguous pixels exceeding 1 mm² with CT attenuation values over 130 Hounsfield units (HU). The Agatston score was then computed as the weighted sum of these calcified plaque areas, adjusted according to their maximum attenuation values [13]. For consistency, the calcium scoring was based on the total Agatston score derived from ECG-gated calcium CT scans with a 3.0 mm slice thickness. The performance of DL-CACS for both ECG-gated calcium CT and non-gated LDCT in the SNUH dataset was analyzed using only calcium scores from ECG-gated CT as the reference standard. Although technically feasible, generating a manual calcium score for non-gated CT images as the reference was impractical due to the large sample size (652 cases) and substantial number of images generated by the thin slice thicknesses (1 mm or 1.25 mm).

For independent validation using Stanford-COCA dataset, the CACS from the ECG-gated calcium CT served as the reference standard for ECG-gated CT. For non-gated LDCT, two reference standards were applied: the calcium score provided from ECG-gated CT in the Stanford-COCA dataset and a manually calculated calcium score from non-gated LDCT images. This dual-reference approach was designed to identify discrepancies arising from software performance or intrinsic differences between different scan types. The manual CACS on non-gated LDCT images was calculated using CT software program (Rapidia 2.8, INFINITT) by the same technologist, supervised by a radiologist with over 20 years of experience.

To strengthen the reference standard, we assessed inter- and intra-observer variability for non-gated LDCT scans. For inter-observer variability, a second technologist, blinded to initial scores, conducted independent evaluations. Intra-observer variability was assessed by having the primary technologist re-score the same cases after a 1-month interval.

### DL-based automated CACS

**Fig. 2:**
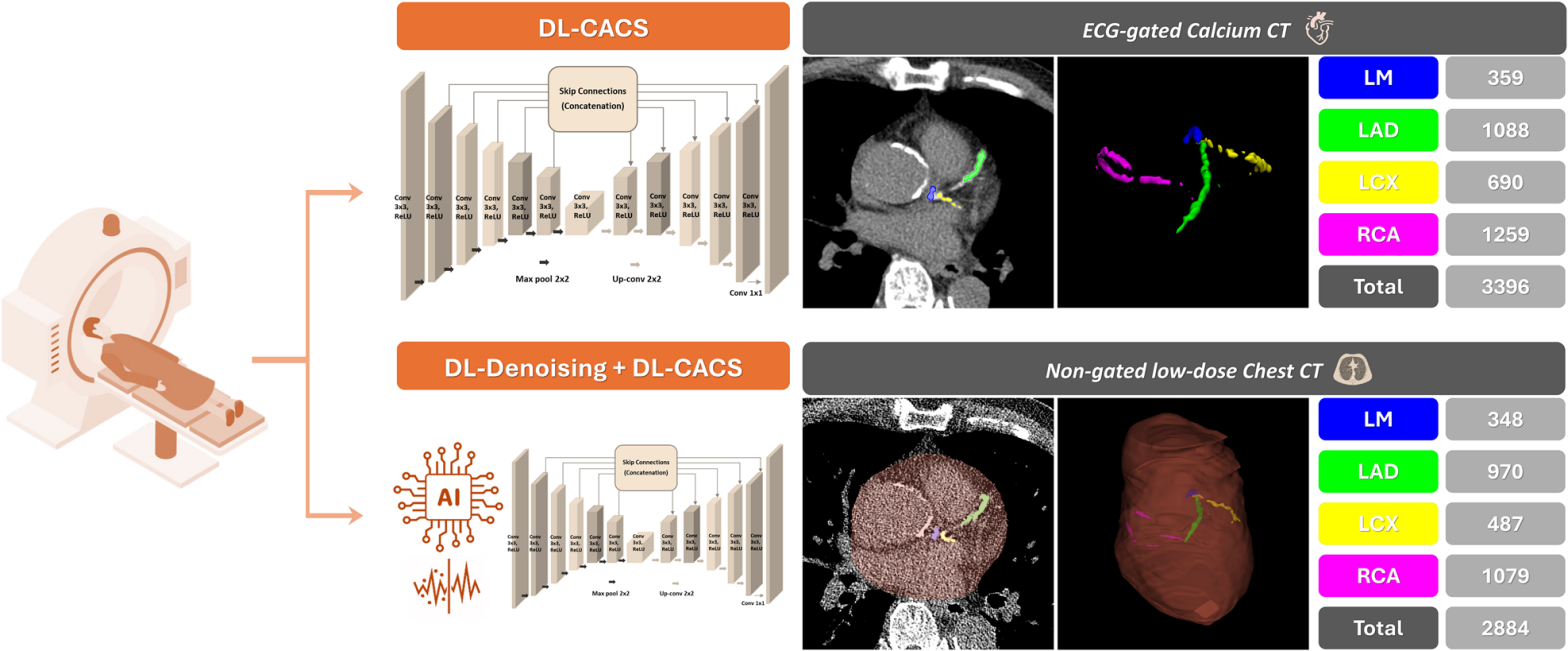
Schematics of the fully automatic deep-learning coronary artery calcium scoring (DL-CACS) software operating on both ECG-gated calcium CT and non-gated low-dose chest CT

Automated DL-CACS was conducted using dedicated software (ClariCardio, ClariPi Inc.) based on a 2D U-net model for ECG-gated calcium CT, which is also compatible with non-gated LDCT data (Fig. 2). DL-based denoising software (ClariCT.AI, ClariPi Inc.) was used to reduce extreme noise in LDCT to a level comparable with standard radiation CT. Data processing was performed on a workstation (Intel Core i9-9900, 128GB RAM, NVIDIA GeForce RTX 3090), requiring approximately 15 s for ECG-gated calcium CT scans and 1.5 min for non-gated LDCT scans per patient. Categorical risk classifications used for predicting future ASCVD events were: 0 (very low), 1–99 (mild), 100–299 (moderate), and ≥ 300 (moderate to severe) [14]. The causes of erroneous categorization in non-gated LDCT, using ECG-gated calcium scores as the reference, were also analyzed. Notably, none of the DL-based software had been trained on the datasets analyzed in this study.

### Statistical analysis

Continuous variables were presented as mean ± standard deviation and categorical variables as absolute numbers with frequencies. Correlations were analyzed using a coefficient of determination (*R*^2^). CAC score agreement was assessed using an interclass correlation coefficient (ICC): poor (< 0.5), fair (0.5–0.75), good (0.75–0.90), and excellent (> 0.90). Categorical agreements of CACS were evaluated using weighted kappa (κ) statistics: none (0.00–0.20), minimal (0.21–0.39), weak (0.40–0.59), moderate (0.60–0.79), strong (0.80–0.90), almost perfect (> 0.90) [15]. The accuracy of categorical risk stratification for the DL-CACS was analyzed. The diagnostic performance of DL-CACS in categorical risk stratification was assessed through both accuracy and area under the receiver operating characteristic curve (AUROC) analysis. Accuracy was calculated for each clinically relevant CAC threshold (0, 100, and 300) to represent the proportion of correct classifications. AUROC was also calculated for these thresholds, with values closer to 1 indicating better discrimination between risk categories. AUROC values > 0.9 were considered excellent, 0.8–0.9 as good, and 0.7–0.8 as fair. Statistical significance was defined as a *p*-value < 0.05. All analyses were conducted using SPSS (version 25.0, IBM Corp.).

## Results

### Evaluation of fully automated DL-CACS in ECG-gated calcium CT

**Fig. 3:**
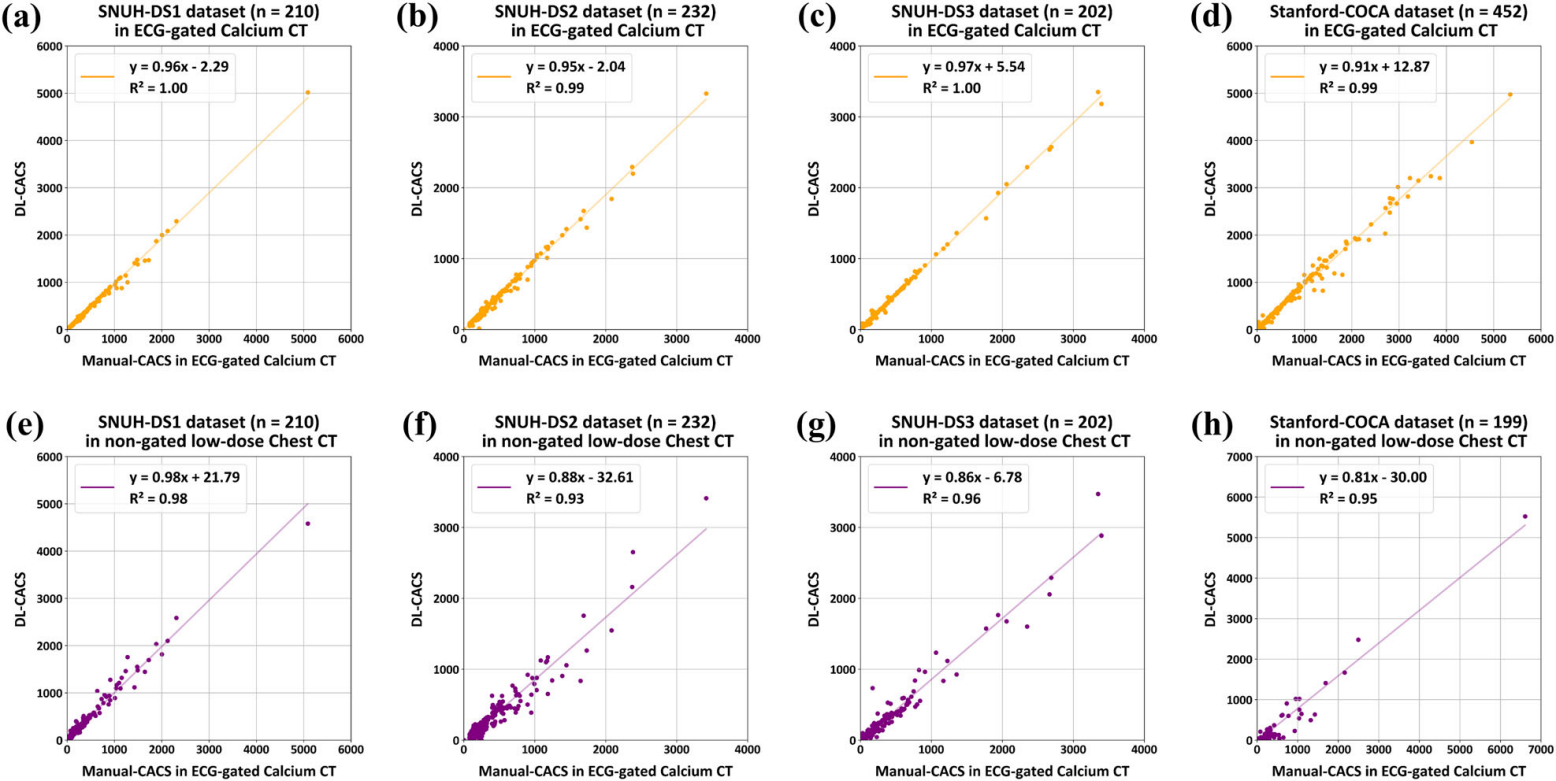
Correlation coefficients between manual coronary artery calcium scores (CACS) and deep-learning-based coronary artery calcium scoring (DL-CACS) in ECG-gated calcium CT and non-gated low-dose chest CT (LDCT). The results in **a**–**d** are based on ECG-gated calcium CT scans from Seoul National University Hospital dataset 1–3 (SNUH-DS1–3) and the Stanford-coronary calcium analysis (Stanford-COCA) dataset, while **e**–**h** show results evaluated on the same datasets obtained from non-gated LDCT scans

Although the calcium score generated by the automated DL-CACS was slightly lower than those of the reference standard, *R*^2^ values indicated a strong correlation in ECG-gated calcium CT data, exceeding 0.99 in both the SNUH and Stanford-COCA datasets (Fig. 3). Agreement between the automated DL-CACS and the reference was excellent across these datasets (Table 2), with categorical risk agreements (*κ*) also reaching almost perfect exceeding 0.95 in both datasets.

**Table 2:** Performance of automated DL-CACS in ECG-gated calcium CT and non-gated low-dose chest CT scans

Acquisition protocol	Dataset	No. of patients	Coefficients of determination, *R*^2^	ICC (95% CI)	Categorical accuracy	Categorical agreement, *κ* (95% CI)
ECG-gated calcium CT	SNUH datasets (overall)	642	0.995	0.997 (0.995–0.997)	96.8%	0.97 (0.96–0.98)
	SNUH-DS1 (GE)	210	0.995	0.997 (0.995–0.998)	98.6%	0.99 (0.97–1.00)
	SNUH-DS2 (Philips)	232	0.992	0.994 (0.989–0.996)	95.3%	0.95 (0.93–0.98)
	SNUH-DS3 (Siemens)	210	0.997	0.998 (0.998–0.999)	96.7%	0.97 (0.95–0.99)
	Stanford dataset: COCA	425	0.989	0.990 (0.988–0.992)	96.7%	0.97 (0.95–0.98)
Non-gated low-dose chest CT	SNUH datasets (overall)	642	0.948	0.968 (0.957–0.976)	86.0%	0.88 (0.85–0.90)
	SNUH-DS1 (GE)	210	0.975	0.987 (0.983–0.990)	93.8%	0.95 (0.92–0.98)
	SNUH-DS2 (Philips)	232	0.926	0.943 (0.857–0.971)	81.0%	0.82 (0.77–0.87)
	SNUH-DS3 (Siemens)	210	0.960	0.970 (0.946–0.982)	85.7%	0.88 (0.83–0.92)
	Stanford dataset: COCA	199	0.949	0.948 (0.921–0.965)	72.4%	0.71 (0.64–0.78)

*DL-CACS* deep-learning-based coronary artery calcium scoring, *ICC* intraclass correlation coefficient, *CI* confidence interval

The DL-CACS achieved categorical accuracies above 95% for ECG-gated calcium CT in both SNUH and Stanford-COCA datasets (Table 2). For diagnostic performance, the AUROC values of DL-CACS in ECG-gated calcium CT were consistently 0.99 for CAC thresholds of 0, 100, and 300 in both the SNUH and Stanford-COCA datasets. These high AUROC values support DL-CACS as a reliable tool for distinguishing cardiovascular risk levels in ECG-gated CT data.

**Fig. 4:**
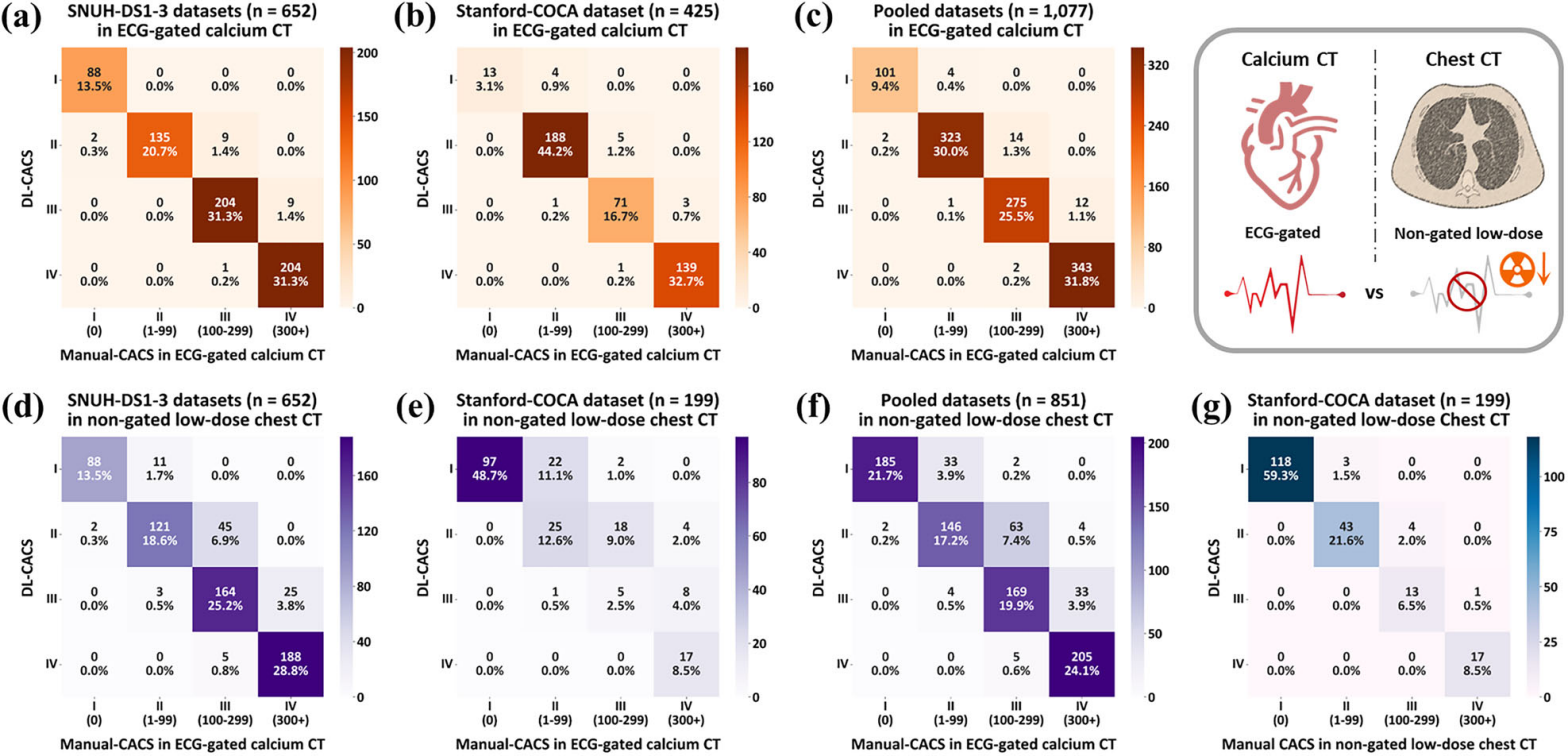
Confusion matrix for categorical risk classification of deep-learning-based coronary artery calcium scoring (DL-CACS) based on total calcium score. **a**–**f** present the confusion matrix comparing DL-CACS to the reference standard from ECG-gated calcium CT, while **g** shows the confusion matrix comparing DL-CACS to the reference standard from non-gated low-dose chest CT. Cardiovascular risk categories are defined as follows: very low (0), mild (1–99), moderate (100–299), and moderate to severe (≥ 300). The side color bar represents the frequency of each risk category

In the pooled data of 1077 cases, 35 (3.2%) cases were misclassified into a different cardiovascular risk category, with the majority (30 out of 35, 85.7%) shifted to a lower-risk category (Fig. 4).

### Evaluation of DL-CACS in non-gated LDCT with manual CACS from non-gated LDCT as the reference

Using manual CACS from non-gated LDCT as the reference, the *R*^2^ for the Stanford-COCA dataset was excellent, with a value of 0.988. Agreement was excellent, with an ICC of 0.994 (95% confidence interval, 0.992–0.995). Categorical accuracy for risk stratification was 96.0% with a weighted *κ* of 0.96.

ROC analysis for non-gated LDCT produced AUROC values of 0.98 for the 0 threshold, 0.99 for 100, and 0.99 for 300, showing strong discriminatory ability in non-gated CT using a non-gated reference. No cases were misclassified into a higher-risk category, though eight cases (4%) were misclassified into lower-risk categories (Fig. 4).

### Evaluation of DL-CACS in non-gated LDCT with manual CACS from ECG-gated calcium CT as the reference

For SNUH datasets, *R*^2^ values ranged from 0.926 to 0.975, with a value of 0.949 for Stanford-COCA dataset, indicating a strong correlation between DL-CACS and reference standard. Agreement was similarly high, with an ICC of 0.968 for the SNUH datasets and 0.948 for Stanford-COCA datasets (Fig. 3 and Table 2). Categorical agreement was excellent, with weighted *κ* values of 0.82–0.94 for the SNUH datasets and moderate agreement with a weighted *κ* of 0.71 for the Stanford-COCA dataset.

Using manual CACS from ECG-gated calcium CT as the reference, the accuracies of the categorical risk classification for DL-CACS in non-gated LDCT ranged from 81.0 to 93.8% for the SNUH datasets and 72.4% in the Stanford-COCA dataset (Table 2). The AUROC values for DL-CACS in non-gated LDCT were 0.99, 0.98, and 0.99 for the CAC thresholds of 0, 100, and 300, respectively, in the SNUH dataset, while AUROC values were 0.89, 0.97, and 0.98 for these same thresholds in the Stanford-COCA dataset, demonstrating good discriminatory performance across risk categories.

In the pooled data of 851 cases, 146 (17.2%) were misclassified into different cardiovascular risk categories, with 135 (92.5%) shifted to a lower-risk category (Table 3 and Fig. 4). Among the cardiovascular disease risk categories, moderate-risk range (100 $\le$ CAC $<$ 300) showed the largest error rate; 70 (29.3%) of 239 participants were misclassified. In contrast, only two (1.1%) of 187 cases with a calcium score of zero were misclassified into an adjacent category.

**Table 3:** Diagnostic performance of automated DL-CACS for categorical risk classification in the pooled data

Acquisition protocol	CAC-DRS category	No. reference standard	No. correct estimation	Categorical accuracy	No. overestimation	No. underestimation
ECG-gated calcium CT (*n* = 1077)	Very low (CAC = 0)	103	101	98.1%	2 (2%)	0 (0%)
	Mild (1 ≤ CAC < 100)	328	323	98.5%	1 (0.3%)	4 (1.2%)
	Moderate (100 ≤ CAC < 300)	291	275	94.5%	2 (0.7%)	14 (4.8%)
	Moderate to Severe (CAC ≥ 300)	355	343	96.6%	0 (0%)	12 (3.4%)
	Total	1077	1042	96.8%	5 (0.5%)	30 (2.8%)
Non-gated low-dose chest CT (*n* = 851)	Very low (CAC = 0)	187	185	98.9%	2 (1.1%)	0 (0%)
	Mild (1 ≤ CAC < 100)	183	146	79.8%	4 (2.2%)	33 (18.0%)
	Moderate (100 ≤ CAC < 300)	239	169	70.7%	5 (2.1%)	65 (27.2%)
	Moderate to Severe (CAC ≥ 300)	242	205	84.7%	0 (0%)	37 (15.3%)
	Total	851	705	82.8%	11 (1.3%)	135 (15.9%)

*CAC-DRS* Coronary Artery Calcium Data and Reporting System

### Causes for misclassification in non-gated LDCT

**Fig. 5:**
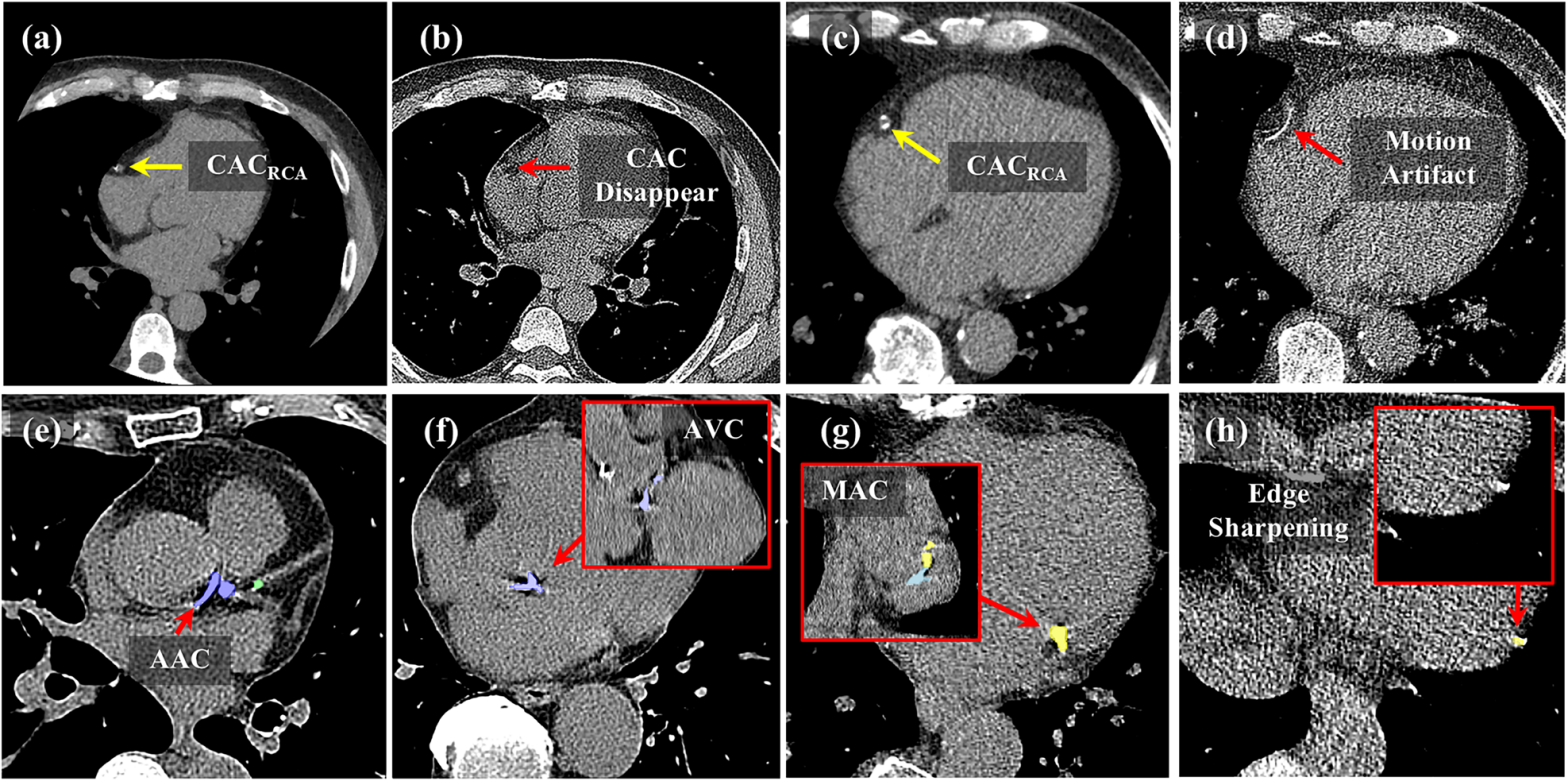
Representative errors in deep-learning-based coronary artery calcium scoring (DL-CACS) on non-gated low-dose chest CT (LDCT). **a**, **b** Information loss for low-density CAC in the right coronary artery (RCA) between ECG-gated calcium CT (yellow arrow) and non-gated LDCT (red arrow) in the same patient. **c**, **d** Motion artifacts for medium- to high-density CAC between two different CT protocols in the same patient (arrows). **e** Ascending aortic calcification (AAC) misclassified as CAC at the left main coronary artery (arrow). **f** Aortic valvular calcification (AVC) incorrectly categorized as CAC (arrow). **g** Mitral annular calcification (MAC) misclassified as CAC at the left circumflex coronary artery (arrow). **h** Edge-sharpening artifact misclassified as CAC (arrow). In the figure, the color overlay represents CAC distribution in each coronary artery blue for the left main, green for the left anterior descending, yellow for the left circumflex, magenta for the right coronary artery (RCA), and sky blue for non-coronary cardiac calcification

In the pooled data of the non-gated LDCT scans from both the SNUH and Stanford-COCA datasets, a total of 146 cases were misclassified when using ECG-gated calcium scores as the reference (Table 3). This included 135 underclassified and 11 overclassified cases. Among the underclassified cases, the primary causes were partial and total CT information loss due to the partial volume effect, accounting for 91 (67.4%) and 23 (17.0%) cases, respectively. Additionally, detection failures due to deep-learning-based model performance limitations resulted in 21 (15.6%) underclassification cases. In the 11 overclassification cases, the most frequent cause was false positives due to motion artifacts (6 cases). Other causes included ascending aortic calcification (1 case), aortic valvular calcification (1 case), mitral annular calcification (1 case), and hallucinated calcification due to the sharpening of the cardiac boundary edge (2 cases) (Fig. 5).

### Inter- and intra-observer variability analysis for manual reference standard

The inter- and intra-observer variability analysis demonstrated excellent agreement for CACS in non-gated LDCT scans. For inter-observer variability, ICC was 0.996, indicating high consistency between independent observers. Intra-observer agreement was similarly strong, with an ICC value of 0.998, reflecting consistent scoring by the primary technologist over time.

## Discussion

The principal findings are as follows. (a) The automated DL-CACS model applied to ECG-gated calcium CT exhibited near-perfect performance (ICC: 0.990, categorical accuracy: 96.7%, and κ: 0.97) in the public validation dataset (Stanford-COCA), with AUROC values of 0.99 for CAC thresholds of 0, 100, and 300. (b) When using the manual calcium score from non-gated LDCT as a reference, the DL-CACS model demonstrated excellent performance with an ICC of 0.994, a categorical accuracy of 96.0%, a κ value of 0.96, and AUROC values of 0.98, 0.99, and 0.99 for the same thresholds in the public validation dataset. (c) When using the manual calcium score from ECG-gated calcium CT as the reference, the DL-CACS model on non-gated LDCT in the public validation dataset demonstrated good performance (ICC: 0.948, categorical accuracy: 72.4%, and κ: 0.71), with AUROC values of 0.89, 0.97, and 0.98 for the thresholds of 0, 100, and 300, respectively.

Recent advancements in artificial intelligence have accelerated research on automated calcium scoring methods for LDCT scans, providing efficient alternatives to the labor-intensive process of manual scoring [7]. Despite this progress, relatively few studies have examined the performance of automated CACS on non-gated LDCT scans across multiple vendor platforms, with reported κ values generally ranging between 0.64 and 0.7 and categorical accuracies from 64 to 82% [16–21]. Notably, these studies have primarily relied on manual CACS from non-gated LDCT as a reference, rather than ECG-gated calcium CT, highlighting the promising performance of fully automated DL-CACS in this study. Lessmann et al [20] developed an automated method for detecting coronary and aortic calcifications in LDCT, facilitating reliable cardiovascular risk assessment in lung cancer screenings. Zeleznik et al [16] evaluated DL-CACS in combined cohort dataset of 5521 participants with ECG-gated calcium CT and non-gated LDCT, achieving a *κ* as 0.7 and categorical accuracy of 78%. In a single-center study of 263 patients, Assen et al [19] validated DL software, achieving a *κ* of 0.74 and categorical accuracy of 82%. Suh et al [18] used a multi-institutional dataset to assess the categorical risk of automated DL-CACS in LDCT, with a κ value of 0.70.

While prior studies have employed the DL-based approach to automatically extract calcium scores, they have evaluated LDCT scans without incorporating advanced noise reduction techniques [16–21]. In contrast, this study integrates an automated AI-based denoising approach for adaptively reducing quantum noise in LDCT scans prior to DL-CACS application. The DL-CACS model utilized in this study also introduces unique features, calculating CACS exclusively within anatomically relevant regions likely to contain coronary artery calcifications (e.g., within the heart), based on a CAC distribution probability map as prior knowledge. Additionally, to reduce false positives, the DL-CACS model categorizes non-coronary calcifications as a separate class during training. This tailored approach aims to improve the accuracy of CAC predictions within real-world clinical settings.

The performance of the studied DL-CACS model declined when ECG-gated CT calcium scores were used as the reference standard. Specifically, the categorical accuracy for risk stratification decreased to 82.8% when applied to pooled non-gated LDCT data (*n* = 851), resulting in a misclassification rate of 17.2%. This decrease is likely due to inherent differences between ECG-gated and non-gated CT protocols rather than a limitation of the DL-CACS model itself. Few studies have directly compared automatic calcium scoring from LDCT against ECG-gated CT references. Notably, Pieszko et al [17], in a study of 507 LDCT scans, observed a κ value of 0.68 and a categorical accuracy of 64%. Dobrolinska et al [7] analyzed 572 patients with suspected coronary artery disease who underwent LDCT alongside PET for myocardial perfusion imaging, yielding a κ value of 0.58 and a categorical accuracy of 48%. This moderate agreement between CACS from ECG-gated calcium CT and non-gated LDCT scans could present notable challenges for clinical application [22]. Despite these challenges, CACS from LDCT scans remains valuable for identifying patients at higher risk of major adverse cardiac events or mortality [23–27].

The observed underestimation of the calcium score in non-gated LDCT compared to those in ECG-gated calcium CT aligns with previous findings [28–30]. Potential causes of CACS discrepancies include differences in CT imaging protocols, intrinsic factors of non-gated low-dose scanning, and performance limitations of the DL-CACS model. First, for CT imaging protocols, the use of thick slices, lower tube voltages, and variations in the X-ray energy distribution caused by collimation filters can impact the CACS discrepancies [31, 32]. For example, compared to the SNUH dataset with a 1 mm slice thickness, the Stanford-COCA dataset with a thicker 5 mm slice thickness showed lower DL-CACS agreement in non-gated LDCT (Tables 1, 2). Additionally, Siemens’ low tube voltage with a tin filter alters the X-ray energy distribution. The Sa36f kernel, used in ECG-gated calcium CT for kV level effects, was not used in this non-gated LDCT cases, resulting in lower concordance in CAC scores [33]. Second, cardiac motion in non-gated scanning can result in both false positives and false negatives. High-intensity CAC information (e.g., > 400 HU) may be falsely duplicated, inflating the calcium scores. Conversely, low-intensity CAC information (e.g., < 150 HU) might be missed, resulting in lower overall calcium scores. The minimal CAC (1 < CAC < 10) was often undetected, with a loss error of 34.6% in 26 cases. Third, increased quantum noise in LDCT scans leads to false positives of the CAC, affected by factors such as thinner slice thickness, sharper kernels, and reduced radiation dose. For instance, the Siemens dataset scanned with a slice thickness of 1 mm with a sharp kernel and a reduced dose displayed inadequate CAC score estimation. Finally, the DL-CACS model’s performance declines due to CAC lesion under-segmentation in blurry CT images and misclassification of non-coronary findings (e.g., ribs or aortic calcifications) causing false positives. Metallic implants in the cardiovascular system can exacerbate the false positives. Despite the potential underestimation and misclassification of CACS in non-gated LDCT, its prognostic value has been widely recognized in large-scale lung cancer screening studies [6, 23, 27]. Standardizing imaging protocols and incorporating AI-based noise reduction techniques may help mitigate these limitations, ensuring more accurate cardiovascular risk stratification in non-gated CT settings.

This study had limitations. First, we did not quantify CACS in individual coronary arteries. Future studies could benefit from reflecting the CACS of each coronary artery to detect any weaknesses in the DL-CACS model for specific coronary arteries. Second, we did not adapt the Agatston cutoff for varying X-ray tube voltages. For example, we maintained the Agatston cutoff at 130 HU even for low tube voltage CT scans, despite recommendations for adjusting HU values for low tube voltages [34]. Third, we did not exclude mitral annular calcifications. Although relatively uncommon, it has occasionally been misclassified as left circumflex coronary artery calcification using the DL-CACS model. Finally, body mass index, which affects quantum noise near the heart, was not factored into the CACS procedure. Elevated noise levels have been observed in individuals with large body sizes and obesity [35]. Applying body mass index-based adaptive image denoising can improve the categorical accuracy of the DL-CACS for LDCT scans.

DL-CACS is a reliable and automated solution for CACS, showing strong performance in both ECG-gated and non-gated CT modalities. It minimizes reliance on manual scoring, potentially enhancing workflow efficiency and supporting cardiovascular risk stratification. Its successful application in non-gated low-dose CT, particularly where ECG-gated CT is unavailable, highlights its potential for broader clinical adoption.

## Abbreviations

AUROC: Area under the receiver operating characteristic curve
CAC: Coronary artery calcium
CACS: Coronary artery calcium scoring
CI: Confidence interval
DL: Deep learning
DL-CACS: Deep-learning-based coronary artery calcium scoring
HU: Hounsfield units
ICC: Intraclass correlation coefficient
LDCT: Low-dose chest CT
SNUH: Seoul National University Hospital
Stanford-COCA: Stanford coronary calcium and chest CT
